# Idiopathic Gangrena Genitalia in Infancy

**Published:** 1870-11

**Authors:** Jonathan W. Brooks

**Affiliations:** 55 South Clark Street; Chicago


A MONTHLY RECORD OF

*Medicine, Surgery, and the Collateral Sciences.*

EDITED BY J. ADAMS ALLEN, M.D., LL.D.; AND WALTER HAY, M.D.

VOL. XXVII.—NOVEMBER, 1870.—No. 11.

### Original Communications.

ARTICLE I.—*Idiopathic Gangræna Genitalia in Infancy.* By JONATHAN W. BROOKS, M.D., Chicago.

The first case of this formidable disease which came under the observation of the writer, was that of L. H., on Sept. 10th, 1838, in a consultation with the late Prof. Worthington Hooker, of New Haven, Ct., and the late Joseph Peabody, M.D., of Buffalo, N. Y. Both gentlemen, at the time named, were residents of Norwich, Conn.

Prof. H. gave us the following history of the case: L. H., aged two years and one month, has been perfectly healthy up to eight days since, when her mother's attention was attracted by her languor, listlessness, want of appetite, haggard looks, and intense thirst. She was pale, and perspired very freely at night. On the 7th of Sept., the mother noticed that there was constant pruritus of the vulva and that she cried bitterly whenever she urinated, and directed the Prof.'s attention thereto. (Hitherto he had regarded the case as one simply of disordered digestion.) On examination, he found the greater labia swollen, spongy to the touch, of a shiny, leaden hue, with watery vesicles dotted here and there over the internal and external surfaces. On the morning of the 9th, he found her crying out with pain in the parts, and some of the vesicles were ruptured and a dark-colored, offensive fluid exuding therefrom; he also found that night quite an intense fever. He applied a yeast poultice, with finely levigated charcoal intermingled, to the parts, gave bark and wine freely with opiates, hoping to find the little innocent's sufferings assuaged thereby; but, on the contrary, everything was intensified the next morning, and Dr. P. and myself were invited to a consultation at 6 A. M., which was the 10th of September. We found her in intense agony (and that hardly expresses her sufferings), with a pallid, contracted countenance, dry skin, rapid and feeble pulse, sleepless, and desiring to micturate every ten minutes, at each effort she screamed with pain. Several ash-colored spots had appeared, viz., one on the perinæum, one near the fourchette within the vulva, one near the clitoris, and two or three near the nymphæ. The whole external genitalia, the perinæum and anal region were swollen and spongy. It was difficult to touch the inner surface of the labia externa or the nymphæ without a bloody ichor issuing from the part touched. An offensive odor exhaled from the parts. The alvine dejections were not unnatural in appearance, but caused great suffering during their passage. The following was agreed on as the treatment to be pursued: Tinct. Ferri Chloridi, eight drops every four hours; one grain of Quinine in brandy every eight hours; and one and one-half grains Pulv. Doveri *pro re nata*, with a liberal allowance of milk punch, animal broths, etc. And a lotion of Tinct. Ferri Chloridi, ʒss. to ℥j. aqua destillat. 11th, found her much as on the 10th, and the treatment was continued.

The 12th, at our consultation, the sufferings were materially less, but the fetor was intolerable. There was a constant ichorous dirty flow from the vagina, and gangrene was advancing rapidly. With little change she went steadily downward for forty hours, and her sufferings were terminated in a protracted convulsion. At the autopsy, twenty-four hours after death, we found all of the genital organs, three-fourths of the neck of the bladder, and one-third of the lower portion of the rectum and anus, destroyed by gangrene, and both pubic bones much damaged. There was about two ounces of pale urine in the bladder. No blood coagula were found in any part of the body. Nothing else was found that seemed unhealthy; and the examination was very thorough.

On the 28th of the same month Prof. H. was called to see J. H., in the same family, a little boy fourteen months older than L., and brother. Had never been ill from birth, but remarkable for vigor and activity up to ten hours preceding this, when his mother's attention was attracted by his feeble, listless, languid ways and haggard looks. A slight diarrhœa developed during the next three hours, but never became very troublesome. He commenced with Tinct. Ferri Chloridi, eight drops every five hours, bark and wine freely as he could take it. Milk punch *ad libitum.* That night he sweat profusely; after that, each evening there was a harsh dry skin, attended with fever; the urine was scanty, not high colored; the pulse rapid, not full. There was a shrunken, pale state of the surface, without sensible change until the third of October, when he began to complain during micturition. Prof. H. saw him immediately, and Dr. P. and the writer within three hours. At this time the left lateral half of the penis, scrotum and adjacent skin were of a deep, shiny, leaden hue. He was constantly disposed to scratch the parts. We advised a continuance of the tonics; and opiates *pro re nata;* also—

℞. Hydrochloric Acid dilut., - - - - ʒ j.

Aq. distillat., - - - - - - - ℥ ij.

M.

To be used constantly as a lotion to the parts, and to add one or two or even three drams of the acid to the water if it did not cause pain, which was done. This treatment was continued for six days with slight variations, and disease seemed to be held at bay and J. improving. At the close of the sixth day, without apparent cause he manifested much increase of pain in urinating and defecating, and in two hours after the opposite side became involved. In a few hours vesicles appeared on the part, and a universal gangrenous condition of the genital organs rapidly followed. As lotions, Hydrochloric, Sulphuric and Acetic Acids in their turns were used, diluted more or less with water, also Liquor Soda Chlor., also tonics of varied kinds, Chlorate of Potass., etc. The treatment at times seemed to hold the disease in check, yet after three weeks of the most intense suffering, he also passed away in a convulsion.

The autopsy, sixteen hours after death, revealed nothing more than in the case of his sister. The entire genitalia and also a large part of the pubic bones were destroyed.

Was requested to meet Dr. T., an old and highly esteemed practitioner, Sept. 15th, 1849, in the same neighborhood hurriedly to see an infant girl, sixteen months old. This case was a repetition of No. 1, and died on the eighteenth; had never been sick before, nor did a post obiit examination give any further light on the disease.

On Sept. 15th, 1853, was solicited to meet, in consultation, Dr. Connor, of Cincinnati, O., and Dr. Mount, of Cumminsville, in a consultation relating to the case of J. P., a little girl residing near Carthage, Ohio, twenty-three months old. Dr. Mount, the attending physician, reported as follows: The child has had perfect health until the 8th of September; on that day she was noticed as unusually dull, languid and feeble, without appetite, and sweating freely at night, which continued until the 12th, when he found her feverish, skin pale and generally contracted. Having regarded it as possibly a masked intermittent, he had given alterative doses of mercurials and from two to three grains of Quinine daily. During the 13th there was great apparent pruritus of the genitals. He directed a lotion of Carb. Soda in water. On the 14th she was evidently worse. On the 15th we found her feeble, haggard, crying out with pain, pulse one hundred and fifty and feeble, countenance hippocratic, the external genitals swollen, dark, shiny, spongy to the touch, and an offensive, dark, serous fluid flowing from the vagina.

The treatment adopted at this time was Tinct. Ferri Chloridi, ten drops every four hours, Sulph. Quinine, milk punch made with brandy *ad libitum*, and any nutriment else that the stomach would tolerate, also a lotion of equal parts of Sulphuric Acid and water. Treatment, however, was of no avail, and she died in a convulsion on the 17th. An autopsy revealed the parts in much the same condition as the first case related. In forty hours from death the whole body was very putrid.

Within the last month the writer has been in attendance on a similar case. The history, so far, may thus be summed up: M. N., aged two years and three months, never had been ill till September 14th from her birth, then her mother noticed her as tired and feeble, looking dull, but did not give much attention to it until the 21st, when with pruritus vulva she also cried out with pain in urinating. On the 22nd the writer saw her for the first time. She had a rapid, irritable pulse, some fever at night, thirst urgent, the genitals swollen, of a leaden hue, with four or five small vesicles on different parts. Directed a diet of milk punch exclusively.

℞. Hyd. Cum Creta, - - - - - - - grs. vj.
Pulv. Rhei, - - - - - - - - - grs. iss.
Pulv. Doveri, - - - - - - - - - grs. ij.
Sacch. Lactis, - - - - - - - - grs. jv.
M. Ft. chart., No. xij. Give one every four hours.

℞. Tinct. Ferri Sesquichloridi, - - - - ℥ j.
S. Give four drops in water once in four hours.

Also take equal parts Tinct. Ferri Sesquichloridi and pure Glycerine, and moisten a piece of soft linen with it and keep constantly applied between the labia, likewise a piece of linen several folds thick, moistened with the same and laid over the parts, changing frequently.

℞. Solution Citrate Potassa, - - - - ℥ ij.
S. Put one teaspoonful in a tumbler of water; use as a drink *ad libitum.*

At my visit the next day, the mother informed me that the pruritus ceased with the first application of the mixture. The vesicles had given place to excavated ulcers, and the mother informed me that a dark colored, gangrenous piece came out of each of them on the previous night, and that she had saved one. There was evidently no ground lost in the case. Directed a continuance of treatment, and bathing the parts with tepid water and Castile soap. On the next day the pulse was slower, the countenance less haggard, prostration slightly less, and the ulcers certainly no worse. Continue treatment. The next day there was improvement, and perhaps half a dozen healthy granulations had appeared. This treatment was continued to Oct. 8th, when the last ulcer was healed. Now (Oct. 12th) she seems pretty well, although pale and feeble. If she recovers fully, Mr. Editor, you shall be informed of it, as it will be the first recovery I have seen from this disease.

It will be observed that this fearfully fatal disease declared itself without apparent cause in perfectly healthy children, all but one were little girls, and all were between one and four years of age. Neither of them had suffered from fever or eruptive disease of any kind, nor was any epidemic of any kind prevalent, or declared itself soon after, except in a single instance, and that was in 1849 when a form of choleroid disease prevailed, and that only among adults. The parents of all the children were very healthy. A singular coincidence was the commencement of each case in the month of September. I have seen partial gangrene of the genitals succeeding to measles, scarlatina, diphtheria and typhoid fever, but at the autopsy emboli were found in the supplying arteries of the parts. But there was nothing of the kind found in either of these cases; on the contrary, however, great fluidity and putrescence of the blood. With one exception, every case has proved fatal, and that I do not regard as exempt from danger. These cases seem to have been induced by a septic poison *sui generis.* Perhaps this will stimulate some one who has had a more successful experience in this direful malady to present their treatment to the profession.

55 South Clark Street.

